# Force control deficits in rapid eye movement behavior disorder and Parkinson’s disease

**DOI:** 10.1016/j.clinph.2025.2110763

**Published:** 2025-05-25

**Authors:** Emily R. Tobin, Stefan Delmas, Joongsuk J. Kim, Jessica C. Hubbard, Basma Yacoubi, XiangYang Lou, Michael F. Presti, Allison Kraus, Richard B. Berry, Michael S. Jaffee, Evangelos A. Christou, David E. Vaillancourt

**Affiliations:** a Department of Applied Physiology and Kinesiology, University of Florida, Gainesville, FL 32611, USA; b Department of Biomedical Engineering, University of Florida, Gainesville, FL 32610, USA; c Department of Biostatistics, University of Florida, Gainesville, FL 32610, USA; d Department of Medicine, Division of Pulmonary, Critical Care and Sleep, University of Florida, Gainesville, FL 32610, USA; e Department of Pathology, Case Western Reserve University School of Medicine, Cleveland, OH 44106, USA; f Department of Neurology, University of Florida, Gainesville, FL 32610, USA

**Keywords:** REM sleep behavior disorder, Parkinson’s disease, Force control, Force variability, ballistic task

## Abstract

**Objective:**

This study aimed to determine if there are deficits in force variability, force increase, force decrease and force errors in rapid eye movement behavior disorder (RBD) using established force control paradigms.

**Methods:**

A cohort of 27 controls, 37 RBD and 37 early-stage Parkinson’s disease (PD) were investigated. Individuals completed constant force and ballistic force control for the finger and ankle.

**Results:**

There was greater force variability in RBD compared with controls and PD during the constant force tasks (p < 0.05). Additionally, we split the RBD group into those with mild and moderate motor impairments and found both groups had higher force variability compared with controls (p < 0.05). PD were slower at increasing and decreasing force (p < 0.05) and this was not observed in the RBD group.

**Conclusion:**

These findings provide new evidence that force variability may be one of the earliest markers of motor dysfunction in RBD before a subsequent diagnosis of neurological disease.

**Significance:**

These findings provide new and important insights into the motor physiological changes in force control in RBD and PD, which may inform future biomarker studies.

## 1. Introduction

Parkinson’s disease (PD) is the second most common neurodegenerative disease and is associated with motor control deficits such as slowness of movement, stiffness, tremor, and postural instability (Hughes et al., 2002). Although clinical diagnostic criteria for PD require bradykinesia with rest tremor, rigidity or both (Armstrong and Okun, 2020; Tolosa et al., 2021) milder motor symptoms could be present before a PD diagnosis (Skorvanek et al., 2018; Summers et al., 2021) and many nonmotor prodromal features are recognized. Of these prodromal features, the one most highly predictive of a subsequent PD diagnosis is Rapid Eye Movement (REM) Behavior Disorder (RBD) (Al-Qassabi et al., 2017; Postuma et al., 2012; Skorvanek et al., 2018). More than 90 % of individuals with idiopathic RBD will eventually develop a synuclein-related neurodegenerative disease, most commonly PD or related conditions such as dementia with Lewy bodies or multiple system atrophy (Galbiati et al., 2019), and there are many unanswered questions as to which types of motor deficits exist in patients with RBD prior to such phenoconversion (Dauvilliers et al., 2018; Doppler et al., 2017; Howell and Schenck, 2015; Hu, 2020). Here, we use established force control paradigms at the index finger and ankle to understand if force variability, force increase, force decrease, force errors, and other measures are impaired in RBD and PD.

In this paper, force control refers to the generation of precise and stable force to a constant target force over time, or fast reverse-at-target goal-directed movement to a specific target. Matching a constant force target over time requires steady force control by reducing variability (Enoka and Stuart, 1992; Vaillancourt et al., 2001), whereas fast reverse-at-target goal-directed movements requires an accurate ballistic force contraction where the speed-accuracy tradeoff is important (Christou and Carlton, 2001; Fernandez et al., 2018; Gordon and Ghez, 1987; Monje et al., 2021; Newell and Carlton, 1988). Force control to a constant target is typically measured using force variability which can be defined as the unintentional variation of movement during voluntary contractions (Christou, 2011). During a constant force pinch grip task individuals with PD have greater force variability compared with controls (Stelmach et al., 1989; Vaillancourt et al., 2001). During a ballistic grip force task, individuals with PD have slower rates of force development (Jordan et al., 1992; Neely et al., 2013; Park and Stelmach, 2007; Spraker et al., 2007; Stelmach and Worringham, 1988) and slower force relaxation (Burciu et al., 2016; Neely et al., 2013; Prodoehl et al., 2010a) compared with controls. PD also demonstrate slower rates of force development and force relaxation during ankle dorsiflexion compared with controls (Chung et al., 2023). Paradigms utilizing force control have not been studied in individuals with RBD. Understanding if there are prodromal motor deficits in patients with RBD may be a sign of increased risk for phenoconversion to a PD diagnosis could lead to the development of better biomarkers and more effective treatment options for both RBD and PD.

In this study, we examined force control deficits in controls, RBD, and PD. We will determine if force control deficits are unique to PD, or if there are overlapping deficits of variability, error, and time to peak force and time of force relaxation in RBD. These findings offer new and important information about the motor physiological changes in force variability, force error, time to peak force and time of force relaxation in RBD and PD that may inform further preclinical biomarker studies.

## 2. Methods

### 2.1. Participants

The study included 27 control, 37 RBD, and 37 PD participants. One control and one PD participant did not participate in the ankle force study. Participants were excluded for active seizures, stroke, brain tumor, pacemaker, or neurostimulator. Controls were also excluded if they had a family history of PD or had a previous traumatic brain injury. In addition, controls were excluded if they had a Movement Disorders Society-Unified Parkinson’s Rating Scale part 3 (MDS-UPDRS-III) score greater than 8. PD participants were diagnosed by a movement disorder specialist at the Fixel Institute for Neurological Disease at the University of Florida. PD participants were ≤ 2 on the Hoehn and Yahr stage when on medication. RBD participants were referred by sleep specialists associated with UF Health and completed a video polysomnography (vPSG). Individuals who met criteria for RBD defined in the International Classification of Sleep Disorders, third edition, text revision (ICSD-3-TR) were enrolled in the study (Sateia, 2014). RBD participants did not meet criteria for any other sleep disorder or movement disorder including but not limited to, narcolepsy, multiple system atrophy, Lewy body dementia, and PD. PD participants who were on antiparkinsonian (dopaminergic) medication performed all testing after overnight withdrawal (>12 h). Controls were recruited by word of mouth and from the surrounding community and were age- and sex-matched to PD and RBD participants. All procedures were approved by the Institutional Review Board and written informed consent was obtained from all participants in accordance with the Declaration of Helsinki.

### 2.2. Clinical assessment

Cognitive screening was assessed by the Montreal Cognitive Assessment (MOCA) (Nasreddine et al., 2005). Participants were administered the MDS-UPDRS-III to classify motor function (Goetz et al., 2008). Bradykinesia was derived from the MDS-UPDRS-III score using items 23–26, which has been done in previous work (Prodoehl et al., 2010b; Stebbins et al., 1999). The REM sleep behavior disorder questionnaire (RBDSQ) was used to further assess and classify RBD (Stiasny-Kolster et al., 2007).

### 2.3. Experimental approach

Participants completed one testing session that lasted ~ 1.5 h in which they performed both the finger and ankle force control studies. Finger and ankle testing were completed in a counterbalanced order. All testing occurred on the non-dominant side, which was determined by the opposite hand they write with (Delmas et al., 2021; Moon et al., 2014; Sainburg, 2002). The finger and ankle force studies included: 1) 3–5 trials of the maximum voluntary contraction (MVC) task, 2) a practice 30 s trial of the constant force task, 3) three 120 s trials of the constant force task, 4) practice 3–5 trials of the goal-directed force task, and 5) 50 trials of the goal-directed force task.

### 2.4. Experimental set-up

Participants were seated comfortably in the upright position and faced a 32-inch monitor (Sync Master 275 t+, Samsung Electronics America) located 1.45 m away at eye level. The monitor was used to display the force produced by index finger abduction and ankle dorsiflexion. All participants affirmed that they could see the display clearly. We used the same setup described in previous literature for the finger force study (Moon et al., 2014) and ankle force study (Casamento-Moran et al., 2020; Delmas et al., 2021).

### 2.5. Force and EMG measurements

During both the finger and ankle force studies, force was recorded consistent with prior work (Casamento-Moran et al., 2020; Delmas et al., 2021; Moon et al., 2014). Muscle activity was recorded with a Trigno wireless electromyography (EMG) sensor (Delsys, Boston, MA) from the first dorsal interosseous (FDI) muscle during finger abduction (Moon et al., 2014) and the tibialis anterior (TA) muscle during ankle dorsiflexion (Casamento-Moran et al., 2020; Delmas et al., 2021).

### 2.6. Maximum voluntary contraction task

The MVC for finger abduction and ankle dorsiflexion were measured by the same methods used in prior work (Delmas et al., 2021; Moon et al., 2014). The best MVC trial was chosen to normalize force for the constant and goal-directed force tasks.

### 2.7. Constant force task

**Fig. 1.**
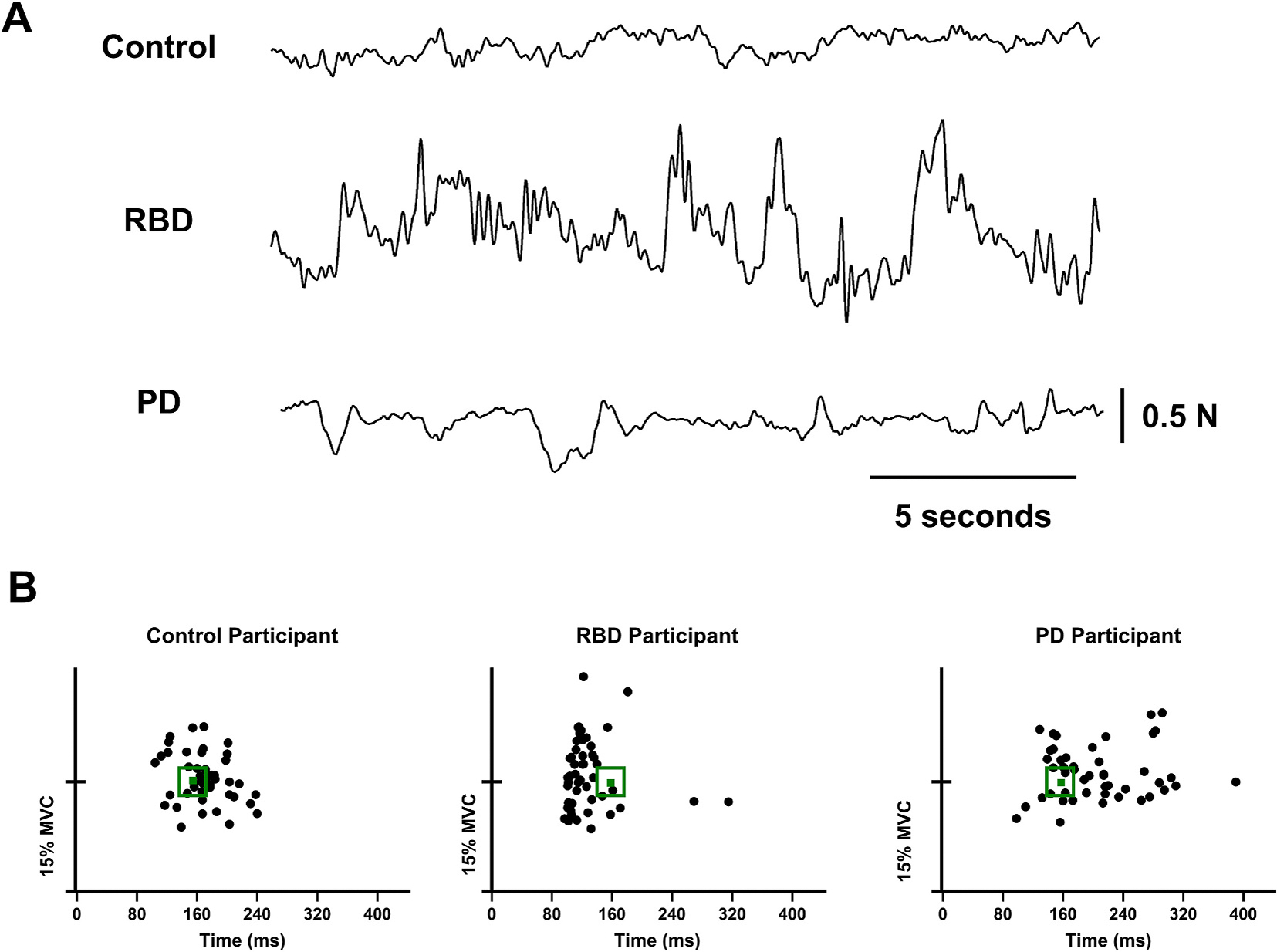
Constant and Goal-Directed Task. **A)** Force signal of one control participant, RBD participant, and PD participant during the constant force task. **B)** All trials of the goal-directed force task for one control participant, RBD participant, and PD participant. Target force and time is represented by the green dot. Abbreviations: PD = Parkinson’s disease; RBD = Rapid Eye Movement Behavior Disorder. (For interpretation of the references to colour in this figure legend, the reader is referred to the web version of this article.)

Participants performed index finger abduction and ankle dorsiflexion against the force sensor (Moon et al., 2014). The target force was set at 15 % MVC and the visual angle was kept constant at 0.01° (Vaillancourt et al., 2006). Participants performed one practice trial lasting 30 s. Then, participants performed 3 trials each lasting 120 s. Participants had at least 30 s of rest between trials. Fig. 1A depicts the force signal between 20–40 s from one control, RBD, and PD during the constant force task. A MATLAB (Math Works Inc., Natick, Massachusetts) program was written and used to manipulate the target force-level and visual feedback.

### 2.8. Goal-directed force task

Participants performed unloaded, fast, reverse-at-target goal-directed movements that involved accurately matching the force to the target (Delmas et al., 2021). Participants had to reverse finger abduction with finger adduction (during the finger force study) and reverse ankle dorsiflexion with ankle plantarflexion (during the ankle force study) at the spatiotemporal target. Participants were given 3–5 practice trials with the goal target set at 25 % MVC and 220 ms. Then, participants performed a total of 50 trials with the goal target set at 15 % MVC and 160 ms. These trials were broken up into 5 blocks of 10 trials. Rest was given between blocks, as needed. Fig. 1B depicts all trials of the goal-directed force task for one control, RBD, and PD participant. A program written in MATLAB manipulated the target force-level and visual feedback.

### 2.9. Data preprocessing

The ankle and finger constant force tasks were each preprocessed using MATLAB. The data was analyzed from 20 to 40 s, and we quantified mean force, coefficient of variation (CV) of force, and root mean square error (RMSE) of force. We calculated the CV to determine the total force variability relative to the mean force. Higher values of CV indicate increased variability of force (Coombes et al., 2010). We calculated the RMSE to measure the deviation of the participants total force output from the target force. Higher values of RMSE indicate less accuracy of force output (Patel et al., 2019). Then, for each participant, the median of each variable was taken across all 3 trials.

The ankle and finger goal-directed tasks were each preprocessed using MATLAB. We quantified peak force, time to peak of force, time to relaxation of force, absolute error of force, absolute error of time, relative error of force, relative error of time, and EMG burst for each trial (Casamento-Moran et al., 2020; Delmas et al., 2021). Then, for each participant, the median of each variable was taken across all 50 trials.

### 2.10. Statistical analysis

All statistical tests were performed in IBM SPSS version 29. Two-tailed tests were performed, where applicable. Descriptive statistics on demographic characteristics and clinical outcomes were calculated as the mean ± standard deviation (SD) for continuous variables and as counts for categorical variables. To evaluate differences between controls, RBD, and PD participants on age, MOCA, MDS-UPDRS-III, MDS-UPDRS-III-Bradykinesia, and RBDSQ we performed one-way analyses of variance (ANOVA). Fisher’s exact test was performed to detect the difference in sex and handedness across groups. For each dependent variable, we tested the homogeneity of group variances using Levene’s test. Depending on the results of the Levene’s test, group differences were analyzed using either parametric analysis of covariances (ANCOVA) or non-parametric analysis (Kruskal-Wallis rank sum test). Age and sex were used as covariates in the ANCOVA model. Pairwise post-hoc comparisons (ANOVA for parametric analysis and Wilcoxon rank sum test for non-parametric analysis) for each task were separately corrected for multiple comparisons (Tukey for parametric analysis and Bonferroni correction Wilcoxon rank sum test) and considered significant when p-_Tukey/Bonferroni_ ≤ 0.05.

As a secondary analysis, we split the RBD group into two based on MDS-UPDRS-III score. RBD participants who scored < 9 on the MDS-UPDRS-III were placed in one group (RBD_Mild). The second group of RBD participants scored ≥ 9 on the MDS-UPDRS-III (RBD_Moderate). The groups for this secondary analysis included 27 controls, 19 RBD_Mild, and 18 RBD_Moderate participants. The same statistical analysis was used for the demographic data, MVC task, constant force task, and goal-directed force task for the finger and ankle studies that was used in the primary analysis.

To observe the relationship between MDS-UPDRS-III-Bradykinesia sub-score and movement quality (CV of force and RMSE of force) we performed non-parametric Spearman’s rho correlations. We performed a total of eight correlations. For the finger force study, we performed a total of four correlations, two for RBD cohort and two for the PD cohort. The same correlations were performed for the ankle force study.

## 3. Results

### 3.1. Demographics

#### 3.1.1. Finger force study

There were no significant differences in age [F(2,98) = 0.526, p = 0.593] or effect of sex among groups (p = 0.561) (Table 1). There were no differences in MOCA score [F(2,98) = 2.927, p = 0.058] or effect of handedness (p = 0.311) among groups. There was a significant difference in MDS-UPDRS-III score [F(2,98) = 25.55, p < 0.001], MDS-UPDRS-III-Bradykinesia subscore [F(2,98) = 15.31, p < 0.001], and RBDSQ score [F(2,98) = 84.61, p < 0.001] across groups.

In post-hoc analysis, PD had significantly higher MDS-UPDRS-III scores compared with controls and RBD (Table 1). RBD had significantly lower MDS-UPDRS-III scores compared with PD. Controls had a significantly lower MDS-UPDRS-III-Bradykinesia sub-score compared with RBD and PD. RBD had significantly higher RBDSQ scores compared with PD and controls. Controls had significantly lower RBDSQ scores compared with PD.

#### 3.1.2. Ankle force study

There was no significant difference in age [F(2,96) = 0.632, p = 0.534] and sex (p = 0.681) among groups (Table 1). There was not a statistically significant difference in MOCA score [F(2,96) = 2.589, p = 0.080] or handedness (p = 0.291) among groups. There was a significant difference in MDS-UPDRS-III score [F(2,96) = 23.89, p < 0.001], MDS-UPDRS-III-Bradykinesia subscore [F(2,96) = 14.76, p < 0.001], and RBDSQ score [F(2,96) = 86.29, p < 0.001] across groups.

In post-hoc analysis, PD had significantly higher MDS-UPDRS-III scores compared with controls and RBD (Table 1). Controls had significantly lower MDS-UPDRS-III scores compared with RBD. Controls had a significantly lower MDS-UPDRS-III-Bradykinesia sub-score compared with RBD and PD. RBD had significantly lower UPDRS-III-Bradykinesia sub-score compared with PD. RBD had significantly higher RBDSQ scores compared with PD and controls. PD had significantly higher RBDSQ scores compared with controls.

### 3.2. Maximal voluntary contraction task

#### 3.2.1. Finger force study

There was a significant difference in MVC across groups [F(2,96) = 5.469, p = 0.006] (Table 2). In post hoc analysis, individuals with PD had significantly lower MVC compared with controls.

#### 3.2.2. Ankle force study

There was no significant difference in MVC among groups [F(2,94) = 1.898, p = 0.156] (Table 2).

### 3.3. Constant force task

#### 3.3.1. Finger force study

The difference in mean force between the rank totals of 64.67 (controls), 46.92 (RBD) and 45.11 (PD) was significant, [H(2, n = 101) = 8.088, p = 0.018] (Table 2). The difference in CV of force between the rank totals of 31.30 (controls), 62.32 (RBD) and 54.05 (PD) was significant, [H(2, n = 101) = 18.14, p < 0.001]. The difference in RMSE of force between the rank totals of 41.96 (controls), 59.73 (RBD) and 48.86 (PD) was significant, [H(2, n = 101) = 6.049, p = 0.049].

**Fig. 2.**
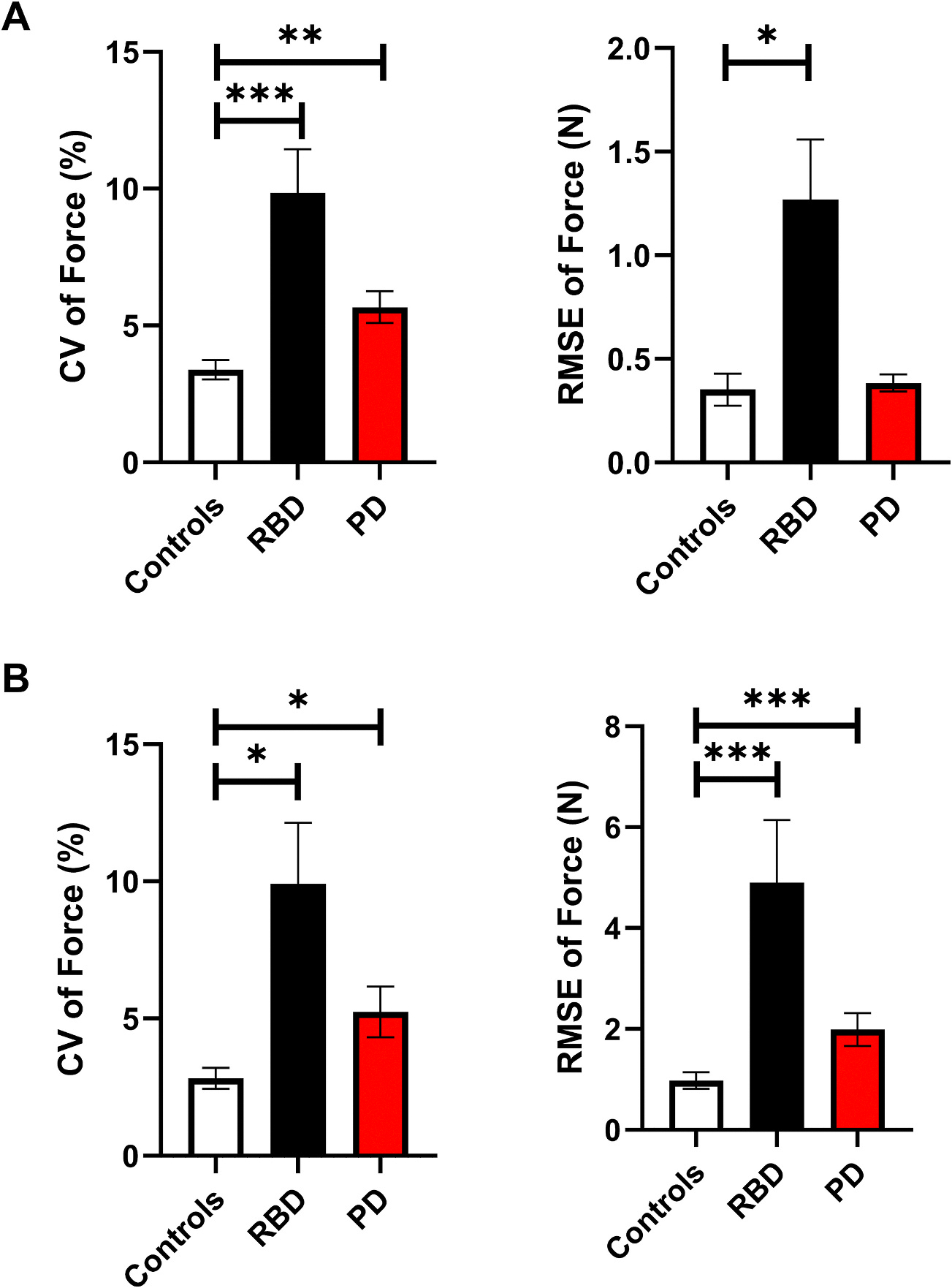
Constant Force Task. **A) Finger Force Study.** Group means (± 1 SEM) for the CV of force and RMSE of force. **B) Ankle Force Study.** Group means (± 1 SEM) for the CV of force and RMSE of force. Significance indicated by * p-_Bonferroni_ < 0.05, ** p-_Bonferroni_ < 0.01, and *** p-_Bonferroni_ < 0.001. Note: Kruskal-Wallis Test was used. Abbreviations: CV = coefficient of variation; PD = Parkinson’s disease; RBD = Rapid Eye Movement Behavior Disorder; RMSE = root mean squared error; SEM = standard error of the mean.

CV of force and RMSE of force are depicted in Fig. 2A. In post-hoc analysis, we found that controls had significantly higher mean force compared with PD and RBD (Table 2). Controls had a significantly smaller CV of force compared with PD and RBD. RBD had a significantly larger RMSE of force compared with controls.

#### 3.3.2. Ankle force study

The difference in mean force between the rank totals of 53.85 (controls), 50.39 (RBD) and 46.92 (PD) was not significant, [H(2, n = 99) = 0.899, p = 0.638] (Table 2). The difference in CV of force between the rank totals of 36.54 (controls), 54.69 (RBD) and 54.89 (PD) was significant, [H(2, n = 99) = 7.746, p = 0.021]. The difference in RMSE of force between the rank totals of 30.54 (controls), 56.81 (RBD) and 57.05 (PD) was significant, [H(2, n = 99) = 16.19, p < 0.001].

CV of force and RMSE of force are depicted in Fig. 2B. In post-hoc analysis, we found that controls had significantly lower CV of force compared with RBD and PD (Table 2). Controls had significantly lower RMSE of force compared with RBD and PD.

### 3.4. Goal-directed force task

#### 3.4.1. Finger force study

There was a significant difference in peak force [F(2,96) = 5.486, p = 0.006], time to peak of force [F(2,96) = 8.408, p < 0.001], time to relaxation of force [F(2,96) = 7.583, p < 0.001], relative error of time [F(2,96) = 6.748, p = 0.002], and EMG burst of the FDI muscle [F(2,96) = 3.409, p = 0.037] across groups (Table 3). There was not a significant difference in absolute error of force [F(2,96) = 2.738, p = 0.070], absolute error of time [F(2,96) = 2.850, p = 0.063], and relative error of force [F(2,96) = 0.154, p = 0.858] among groups.

**Fig. 3.**
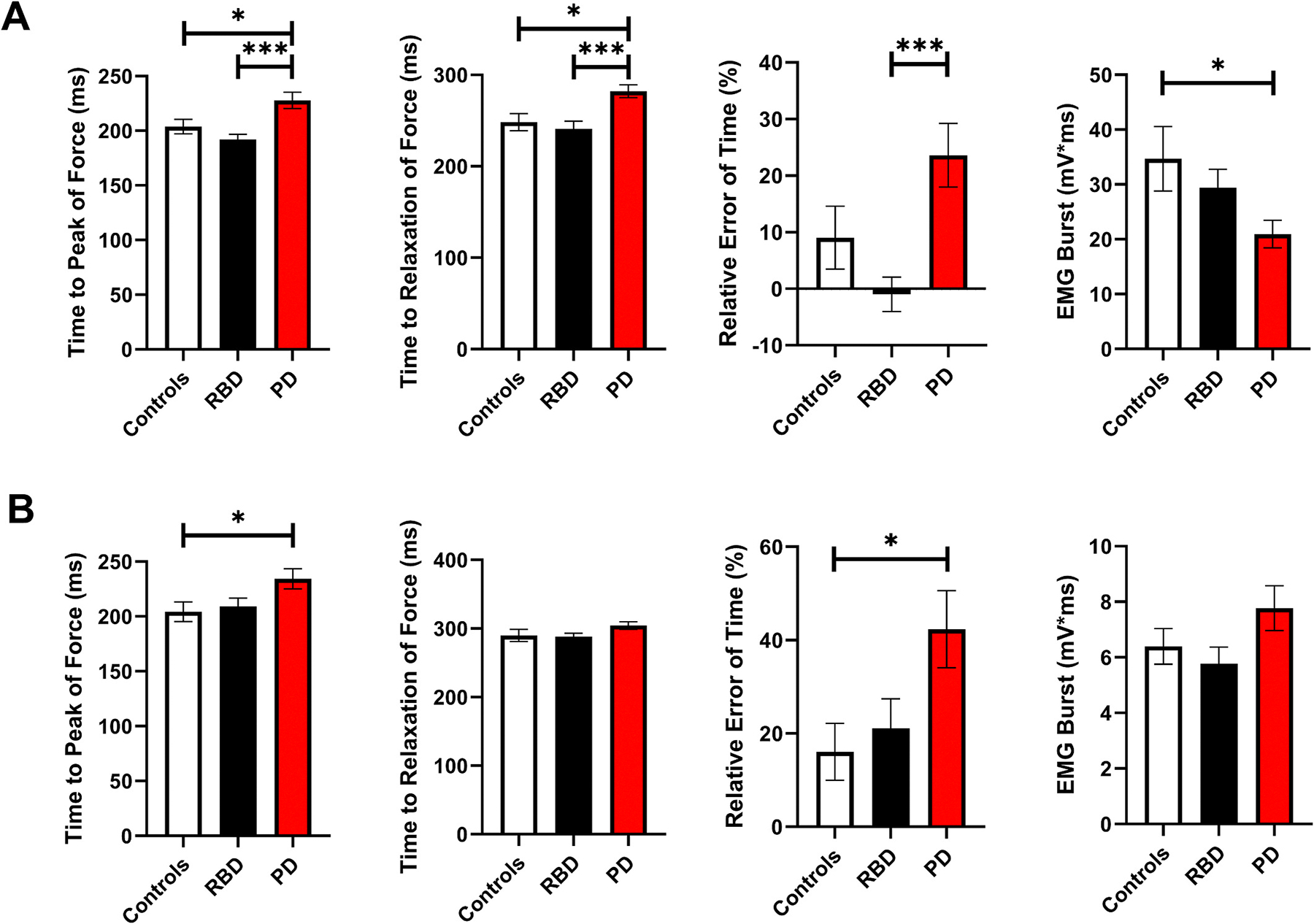
Goal-Directed Force Task. A) Finger Force Study. Group means (± 1 SEM) for the Time to Relaxation of Force, Time to Peak of Force, Relative Error of Time, and EMG Burst of the FDI muscle. **B) Ankle Force Study.** Group means (± 1 SEM) for the Time to Relaxation of Force, Time to Peak of Force, Relative Error of Time, and EMG Burst of the TA muscle. Significance indicated by * p-_Tukey_ < 0.05, ** p-_Tukey_ < 0.01, and *** p-_Tukey_ < 0.001. Note: ANCOVA covarying for age and sex was used. Abbreviations: PD = Parkinson’s disease; RBD = Rapid Eye Movement Behavior Disorder; RMSE = root mean squared error; SEM = standard error of the mean.

Time to relaxation of force, time to peak of force, relative error of time, and EMG burst of the FDI muscle are depicted in Fig. 3A. In post-hoc analysis, controls had a significantly larger peak force compared with PD (Table 3). PD had a significantly longer time to peak of force compared with controls and RBD. PD had a significantly longer time to relaxation of force compared with controls and RBD. PD had a significantly larger relative error of time compared with RBD. PD had a significantly smaller EMG burst of the FDI muscle compared with controls.

#### 3.4.2. Ankle force study

There was a significant difference in time to peak of force [F(2,94) = 3.832, p = 0.025] and relative error of time [F(2,94) = 3.985, p = 0.022] across groups (Table 3). There was not a significant difference in peak force [F(2,94) = 0.481, p = 0.619], time to relaxation of force [F(2,94) = 2.236, p = 0.113], absolute error of force [F(2,94) = 0.953, p = 0.389], absolute error of time [F(2,94) = 3.050, p = 0.052], relative error of force [F(2,94) = 1.197, p = 0.307], and EMG burst of the TA muscle [F(2,94) = 2.310, p = 0.105] among groups.

Time to relaxation of force, time to peak of force, relative error of time, and EMG burst of the TA muscle are depicted in Fig. 3B. In post-hoc analysis, PD had significantly longer time to peak of force compared with controls (Table 3). PD had a significantly larger relative error of time compared with controls.

### 3.5. RBD_Mild and RBD_Moderate cohort

#### 3.5.1. Demographics

##### 3.5.1.1. Finger force study.

Demographic statistics for controls, RBD_Mild and RBD_Moderate are summarized in Table 4. There were no significant differences in age [F(2,61) = 1.012,p = 0.370] or effect of sex (p = 0.557) among groups. There were no significant difference in MOCA score [F(2,61) = 2.000, p = 0.144] or significant effect of handedness (p = 0.073) among groups. We found a difference in MDS-UPDRS-III score [F(2,61) = 46.77, p < 0.001] and RBDSQ score [F (2,61) = 73.73, p < 0.001] across groups. In post-hoc analysis we found that RBD_Moderate had significantly higher MDS-UPDRS-III scores compared with controls and RBD_Mild. Controls had significantly lower RBDSQ scores compared with RBD_Mild and RBD_Moderate.

##### 3.5.1.2. Ankle force study.

Demographic statistics for controls, RBD_Mild and RBD_Moderate are summarized in Table 4. There was not a significant difference in age [F(2,60) = 1.181, p = 0.314] or a significant effect of sex (p = 0.613) among groups. There was not a statistically significant MOCA score [F(2,60) = 1.725, p = 0.187] or handedness (p = 0.065) among groups. We found a difference in MDS-UPDRS-III score [F(2,60) = 45.45, p < 0.001] and RBDSQ score [F (2,60) = 70.31, p < 0.001] across groups. In post-hoc analysis, we found that RBD_Moderate had significantly higher MDS-UPDRS-III scores compared with controls and RBD_Mild. Controls had significantly lower RBDSQ scores compared with RBD_Mild and RBD_Moderate.

#### 3.5.2. MVC and constant force tasks

##### 3.5.2.1. Finger force study.

**Fig. 4.**
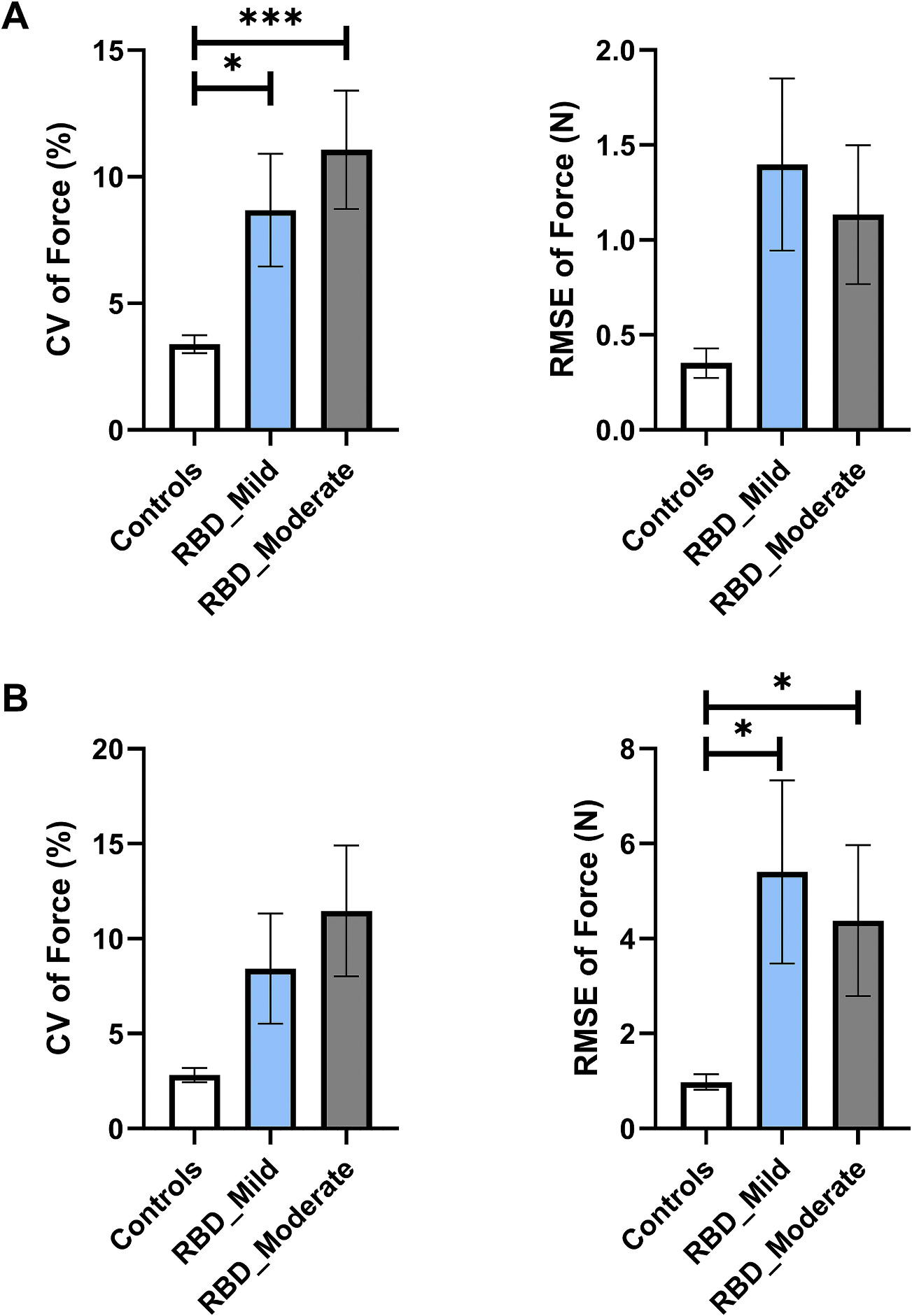
RBD_Mild vs RBD_Moderate - Constant Force Task. **A) Finger Force Study.** Group means (± 1 SEM) for the CV of Force and RMSE of Force. **B) Ankle Force Study.** Group means (± 1 SEM) for the CV of Force and RMSE of Force. Significance indicated by * p-_Bonferroni_ < 0.05, ** p-_Bonferroni_ < 0.01, and *** p-_Bonferroni_ < 0.001. Note: Kruskal-Wallis Test was used. Abbreviations: CV = coefficient of variation; PD = Parkinson’s disease; RBD = Rapid Eye Movement Behavior Disorder; RBD_Mild = RBD MDS-UPDRS score < 9; RBD_Moderate = RBD MDS-UPDRS score ≥ 9; RMSE = root mean squared error; SEM = standard error of the mean.

MVC and constant force tasks findings between controls, RBD_Mild and RBD_Moderate are summarized in Table 5. There was not a significant difference in MVC [F(2,59) = 0.864, p = 0.427] and mean force [F(2,59) = 3.143, p = 0.050] among groups. The difference in CV of force between the rank totals of 21.37 (controls), 37.42 (RBD_Mild), and 44.00 (RBD_Moderate) was significant, [H(2, n = 64) = 17.84, p < 0.001]. The difference in RMSE of force between the rank totals of 26.26 (controls), 35.21 (RBD_Mild), and 39.00 (RBD_Moderate) was not significant, [H(2, n = 64) = 5.630, p = 0.060]. In post-hoc analysis, we found that controls had a significantly lower CV of force compared with RBD_Mild and RBD_Moderate (Fig. 4).

##### 3.5.2.2. Ankle force study.

MVC and constant force task findings between controls, RBD_Mild and RBD_Moderate are summarized in Table 5. There was not a significant difference in MVC among groups [F(2,58) = 1.316, p = 0.846]. The difference in mean force between the rank totals of 34.62 (controls), 28.32 (RBD_Mild) and 32.11 (RBD_Moderate) was not significant, [H(2, n = 63) = 1.298, p = 0.523]. The difference in CV of force between the rank totals of 25.42 (controls), 36.84 (RBD_Mild) and 36.39 (RBD_Moderate) was not significant, [H(2, n = 63) = 5.705, p = 0.058]. The difference in RMSE of force between the rank totals of 22.88 (controls), 38.79 (RBD_Mild) and 38.00 (RBD_Moderate) was significant, [H(2, n = 63) = 10.97, p = 0.004]. In post-hoc analysis, we found that controls had a significantly lower RMSE of force compared with RBD_Mild and RBD_Moderate (Fig. 4).

#### 3.5.3. Goal-directed force task

##### 3.5.3.1. Finger force study.

Goal-directed force task findings between controls, RBD_Mild and RBD_Moderate are summarized in Table 5. We found differences in time to peak of force [F(2,59) = 5.108, p = 0.009] and relative error of time [F(2,59) = 3.780, p = 0.029] across groups. There was not a significant difference in peak force [F(2,59) = 1.917, p = 0.156], time to relaxation of force [F(2,59) = 2.052, p = 0.138], absolute error of force [F(2,59) = 1.100, p = 0.340], absolute error of time [F(2,59) = 0.532, p = 0.590], relative error of force [F(2,59) = 0.095, p = 0.910] and EMG burst of the FDI muscle [F(2,59) = 0.515, p = 0.600] among groups. In post-hoc analysis, we found that RBD_Mild had a significantly shorter time to peak of force compared with controls and RBD_Moderate. Controls had a significantly larger relative error of time compared with RBD_Mild.

##### 3.5.3.2. Ankle force study.

There was not a significant difference in peak force [F(2,58) = 0.781, p = 0.463], time to peak of force [F(2,58) = 1.316, p = 0.276], time to relaxation of force [F(2,58) = 0.044, p = 0.957], absolute error of force [F(2,58) = 1.140, p = 0.327], absolute error of time [F(2,58) = 0.599, p = 0.553], relative error of force [F(2,58) = 0.304, p = 0.739], relative error of time [F(2,58) = 1.459, p = 0.241] and EMG burst of the TA muscle [F(2,58) = 0.225, p = 0.799] among groups.

### 3.6. Correlations between MDS-UPDRS-III-Bradykinesia and movement quality

#### 3.6.1. Finger force study

There was a significant correlation between MDS-UPDRS-III-Bradykinesia and the CV of force for PD [r(37) = 0.335, p = 0.042]. There was not a significant correlation between MDS-UPDRS-III-Bradykinesia and the CV of force for RBD [r(37) = 0.156, p = 0.335]. In addition, there was not a significant correlation between MDS-UPDRS-III-Bradykinesia and the RMSE of force for both RBD [r(37) = 0.151, p = 0.372] and PD [r(37) = 0.280, p = 0.093].

#### 3.6.2. Ankle force study

There was not a significant correlation between MDS-UPDRS-III-Bradykinesia and the CV of force for both RBD [r(37) = −0.062, p = 0.715] and PD [r(36) = 0.131, p = 0.447]. Additionally, there was not a significant correlation between MDS-UPDRS-III-Bradykinesia and the RMSE of force for both RBD [r(37) = −0.056, p = 0.743] and PD [r(36) = −0.095, p = 0.581].

### 3.7. Discussion

We explored force control during upper and lower limb tasks in RBD, early-stage PD, and control participants. We identified that the RBD participants had greater force variability during the finger and ankle force control tasks compared with controls and PD. PD also had increased force variability for the finger and ankle tasks compared with controls. As expected, PD were slower at increasing force and relaxing force, whereas the novel finding here was that RBD were not slower at increasing and decreasing force. This suggests that while RBD participants produce more variable force, they are not bradykinetic at this point in the disease process. In a subgroup analysis, we split the RBD cohort into mild and moderate based on the MDS-UPDRS-III. Both mild and moderate RBD groups showed increased force variability, suggesting that this observation is occurring despite the severity of motor symptoms present. These new findings point to early sensorimotor deficits in force control in RBD and raise some interesting possibilities for nervous system adaptations and speed-accuracy trade-offs.

A novel set of findings in this study was that the RBD group had increased force variability, and normal time to peak force and time of force relaxation. This raises an important question: why would RBD have greater variability? Prior work supports the hypothesis that individuals with PD will slow a movement down in order to perform the motor task accurately (Fernandez et al., 2018; Flowers, 1975; Mazzoni et al., 2007), and this could be evidence that the PD nervous system takes advantage of the well-established speed-accuracy trade-off. It has been previously suggested that this force variability and time to peak force and time of force relaxation are due to noise in the nervous system, which can be from variation in motor planning by central circuits or noise in motor execution that is typically associated with speed-accuracy trade-off (Fitts, 1992; Sutter et al., 2021).

In the current study, we found that RBD had greater variability during isometric force tasks compared with controls and PD. In addition, we split the RBD cohort into two groups based on MDS-UPDRS-III score to differentiate between mild and moderate motor impairments. We found that both RBD groups had significantly higher force variability compared with controls, with no differences between RBD_Mild and RBD_Moderate groups. This suggests that this force variability seen in the RBD cohort is not due to differences in motor impairment. One hypothesis is that increased variability in RBD is related to noise in the central planning circuits. Previous work suggests that the central nervous system accounts for the signal-dependent noise during isometric contractions in movement planning (Jones et al., 2002). This finding points to the hypothesis that variability may be one of the earliest markers of motor dysfunction in RBD before a diagnosis of a movement disorder.

Variability in reaching and goal-directed movements have shown to have both planning and execution noise components (Sutter et al., 2021). Prior work has found that execution noise accounts for a large portion of endpoint variability during reaching tasks (van Beers et al., 2004) and is also associated with the speed-accuracy trade-off (Fitts, 1992). In the current study, we did not see any differences in the time to peak force and time of force relaxation during the goal-directed force tasks in RBD compared with controls, however, we did see differences in PD. One plausible explanation for why we see differences in force variability but no differences in the time to peak force and time of force relaxation in RBD is that they are earlier in the disease process, and they do not have the same level of motor deficits (i.e. bradykinesia) compared with PD. This suggests that individuals with RBD may have motor deficits due to planning noise while PD may have motor deficits due to both planning and execution noise. The dynamic system theory supports this hypothesis by suggesting that variability will increase to a specific critical point, then the system becomes highly unstable and then will switch to a new motor plan that has less variability (Stergiou and Decker, 2011). Previous work suggested that bradykinesia might be an active strategy of the nervous system that individuals with PD develop to achieve accuracy (Sheridan and Flowers, 1990). In addition, it is not known if these individuals with RBD will phenoconvert to PD or another alpha-synuclein disease. It is possible that longitudinally the individuals with RBD will become less variable with reduced rate of force control and further studies will need to address this important point.

In human neuroimaging, it was found that the putamen and superior frontal gyrus are associated with the coefficient of variation of force during an isometric ankle dorsiflexion task (Yoon et al., 2014). In PD, it was found that there is reduced blood-oxygen level-dependent (BOLD) signal in primary motor area (hand area), caudate, putamen, thalamus and cerebellum (lobular V) during isometric grip force paradigm (Burciu et al., 2016; Prodoehl et al., 2010a; Spraker et al., 2010; Tobin et al., 2024). In a longitudinal study of PD, it was found that the motor cortex and putamen had reduced BOLD signal over 12 months suggesting that the neuronal function was degenerating over time (Burciu et al., 2016). In prior work, it was found that individuals with RBD had significantly reduced BOLD signal in the primary motor cortex (hand area), caudate, putamen and thalamus compared with controls during isometric grip force paradigm (Tobin et al., 2024). This suggests that the sensorimotor network may be one of the first brain networks affected before the diagnosis of Parkinsonism. It is not yet clear if specific regions will predict whether an individual with RBD will develop PD, Lewy body dementia or multiple system atrophy, and further work is needed in this area.

Previous work has shown that there is greater force variability in PD compared with controls during constant force tasks using pinch grip (Stelmach et al., 1989; Vaillancourt et al., 2001) and ankle dorsiflexion (Chung et al., 2023; Vaillancourt et al., 2001; Vaillancourt et al., 2001). To characterize force variability the CV of force has been used (Smart et al., 2020). Another important metric that can be quantified during constant force tasks is force accuracy (i.e. RMSE of force) (Patel et al., 2019). The current study found support for prior work, in that PD had a higher variability compared with controls during both constant force tasks and found that PD had less force accuracy compared with controls during the constant ankle force task. Using both metrics, force variability and force accuracy allows us to gain a better understanding of the underlying motor physiology in PD (Yacoubi and Christou, 2024).

There are some limitations of our study. In our study, PD participants did not undergo vPSG sleep study so we could not further classify the PD cohort into those with an RBD diagnosis (PD+RBD) and those without an RBD diagnosis (PD-RBD). Being able to classify the PD cohort into PD+RBD and PD-RBD could allow for a more comprehensive analysis. Another limitation of our study was that the RBD_Mild cohort was younger than the RBD_Moderate cohort. These limitations underscore the need for future studies to follow RBD longitudinally to further understand how motor deficits progress.

### 3.8. Conclusions

In conclusion, the current study revealed that the RBD cohort had greater force variability, and a normal time to peak force and time of force relaxation. This suggests that force variability may be one of the earliest markers of motor dysfunction in RBD. RBD may have a normal time to peak force and time of force relaxation since they have milder motor symptoms than PD and have not developed bradykinesia. In addition, the present study found no differences in force variability in the RBD_Mild and RBD_Moderate cohort compared with controls. This suggests that the force variability in both RBD groups were not related due to motor impairment. Future work will need to study RBD longitudinally to determine if the motor deficits related to force variability, force increase, and force decrease during upper and lower extremity tasks progress over time.

## Figures and Tables

**Table 1:** Participant Demographic Information for the Finger and Ankle Force Studies. Participants sex, handedness, disease duration, age, and mean scores (±1 SD) from the Montreal Cognitive Assessment (MOCA), Movement Disorders Society Unified Parkinson’s Disease Rating Scale Part III (MDS-UPDRS-III), Movement Disorders Society Unified Parkinson’s Disease Rating Scale Part III Bradykinesia subscore (MDS-UPDRS-III-Bradykinesia), and RBD Sleep Questionnaire (RBDSQ). One-way ANOVA and Fisher’s exact test were performed for quantitative and categorical characteristics, respectively, to compare groups.

Finger Force Study	Controls (n = 27)	RBD (n = 37)	PD (n = 37)	p-value

Age (years)	62.85 ± 7.735	61.03 ± 8.742	62.68 ± 7.867	0.593
Sex M / F	14 / 13	25 / 12	22 / 15	0.561
Handedness L / R	6 / 21	3 / 34	5 / 32	0.311
Disease Duration (months)	–	27.31 ± 39.82	29.65 ± 17.47	–
MOCA	26.89 ± 2.501	25.54 ± 2.805	25.43 ± 2.410	0.058
MDS-UPDRS-III	3.519 ± 2.327	10.95 ± 9.315	16.19 ± 6.544	<0.001*** ^a,b,c^
MDS-UPDRS-III-Bradykinesia	2.296 ± 1.772	5.616 ± 4.809	7.730 ± 3.956	<0.001*** ^a,b^
RBDSQ	1.259 ± 1.318	7.459 ± 2.387	2.865 ± 2.043	<0.001*** ^a,b,c^
Ankle Force Study	Controls (n = 26)	RBD (n = 37)	PD (n = 36)	p-value
Age (years)	63.23 ± 7.628	61.03 ± 8.742	62.61 ± 7.969	0.534
Sex M / F	14 / 12	25 / 12	22 / 14	0.681
Handedness L / R	6 / 20	3 / 34	5 / 31	0.253
Disease Duration (months)	–	27.31 ± 39.82	30.14 ± 17.46	–
MOCA	26.81 ± 2.514	25.54 ± 2.805	25.39 ± 2.429	0.080
MDS-UPDRS-III	3.654 ± 2.262	10.95 ± 9.315	16.22 ± 6.634	<0.001*** ^a,b,c^
MDS-UPDRS-III-Bradykinesia	2.385 ± 1.745	5.622 ± 4.809	7.833 ± 3.961	<0.001*** ^a,b,c^
RBDSQ	1.308 ± 1.320	7.459 ± 2.387	2.750 ± 1.948	<0.001*** ^a,b,c^

Significance indicated by *** p < 0.001.

Abbreviations: F = female; MDS-UPDRS-III = Movement Disorders Society - Unified Parkinson’s Disease Rating Scale - Part 3; MDS-UPDRS-III-Bradykinesia = Movement Disorders Society - Unified Parkinson’s Disease Rating Scale - Part 3 Bradykinesia Subscore; L = left; M = male; MOCA = Montreal Cognitive Assessment; PD = Parkinson’s disease; RBD = Rapid Eye Movement Behavior Disorder; RBDSQ = REM Sleep Behavior Disorder Questionnaire; R = right, SD = standard deviation

Pairwise post-hoc analysis when p ≤ 0.05 (Tukey-corrected):

a = Controls versus PD.

b = Controls versus RBD.

c = RBD versus PD.

**Table 2:** **Maximal Voluntary Contraction and Constant Force Tasks for Finger and Ankle Force Studies** - ANCOVAs were run on participants MVC for both the finger and ankle studies. Age and sex were included as covariates. The Kruskal-Wallis rank sum test was run on mean force, CV of force, and RMSE of force (± 1 SD) for the finger and ankle force studies.

Finger Force Study	Controls (n = 27)	RBD (n = 37)	PD (n = 37)	p-value

*Maximum Voluntary Contraction Task*				
MVC	39.57 ± 21.17	35.88 ± 18.52	27.12 ± 12.00	0.006** ^a^
*Constant Force Task*				
Mean Force (N)	5.386 ± 2.422	4.020 ± 3.446	3.917 ± 1.871	0.018* ^a,b^
CV Force (%)	3.391 ± 1.858	9.845 ± 9.736	5.674 ± 3.565	<0.001*** ^a,b^
RMSE Force (N)	0.352 ± 0.402	1.269 ± 1.770	0.384 ± 0.254	0.049* ^b^
Ankle Force Study	Controls (n = 26)	RBD (n = 37)	PD (n = 36)	p-value
*Maximum Voluntary Contraction Task*				
MVC (N)	124.4 ± 46.25	135.1 ± 57.72	115.2 ± 41.37	0.156
*Constant Force Task*				
Mean Force (N)	18.12 ± 6.868	16.10 ± 12.04	16.95 ± 6.160	0.638
CV Force (%)	2.819 ± 1.942	9.903 ± 13.53	5.248 ± 5.545	0.021* ^a,b^
RMSE Force (N)	0.979 ± 0.830	4.904 ± 7.544	1.991 ± 1.949	<0.001*** ^a,b^

Significance indicated by * p < 0.05, ** p < 0.01, and *** p < 0.001.

Abbreviations: CV = coefficient of variation; EMG = electromyography; MVC = maximum voluntary contraction; PD = Parkinson’s disease; RBD = Rapid Eye Movement Behavior Disorder; RMSE = root mean squared error, SD = standard deviation

Pairwise post-hoc analysis when p ≤ 0.05 (MVC Task = Tukey-corrected, Constant Force Task = Bonferroni-corrected):

a = Controls versus PD.

b = Controls versus RBD.

c = RBD versus PD.

**Table 3:** Goal-Directed Force Task for the Finger and Ankle Force Studies. ANCOVAs were run for participants time to relaxation of force, time to peak of force, peak force, absolute error of force, relative error of force, absolute error of time, relative error of time, and EMG burst (± 1 SD) for the ankle and finger force studies. Age and sex were included as covariates.

Finger Force Study	Controls (n = 27)	RBD (n = 37)	PD (n = 37)	F-Statistic	p-value

Peak Force (N)	6.604 ± 3.037	5.999 ± 3.451	4.679 ± 2.023	5.486	0.006** ^a^
Time to Peak of Force (ms)	203.8 ± 34.48	191.9 ± 29.25	227.8 ± 45.96	8.408	< 0.001*** ^a,c^
Time to Relaxation of Force (ms)	248.3 ± 48.82	240.9 ± 51.15	282.1 ± 42.86	7.583	<0.001*** ^a,c^
Absolute Error of Force (N)	1.531 ± 0.784	1.552 ± 1.119	1.136 ± 0.571	2.738	0.070
Absolute Error of Time (ms)	43.39 ± 36.74	37.38 ± 14.74	56.76 ± 45.14	2.850	0.063
Relative Error of Force (%)	19.06 ± 17.16	18.50 ± 20.51	16.59 ± 23.00	0.154	0.858
Relative Error of Time (%)	9.039 ± 28.98	-0.997 ± 18.50	23.59 ± 34.23	6.748	0.002** ^c^
EMG Burst (mV*ms)	34.69 ± 30.50	29.41 ± 20.55	20.95 ± 15.40	3.409	0.037* ^a^
Ankle Force Study	Controls (n = 26)	RBD (n = 37)	PD (n = 36)	F-Statistic	p-value
Peak Force (N)	24.24 ± 9.248	27.95 ± 13.53	25.82 ± 10.19	0.481	0.619
Time to Peak of Force (ms)	204.3 ± 45.73	209.1 ± 46.64	234.3 ± 54.64	3.832	0.025* ^a^
Time to Relaxation of Force (ms)	289.9 ± 44.89	288.3 ± 29.90	304.4 ± 32.63	2.236	0.113
Absolute Error of Force (N)	6.681 ± 4.817	9.05 ± 6.961	8.812 ± 6.911	0.953	0.389
Absolute Error of Time (ms)	46.98 ± 39.50	51.68 ± 52.20	78.57 ± 71.59	3.050	0.052
Relative Error of Force (%)	34.51 ± 34.21	37.79 ± 42.84	49.54 ± 52.40	1.197	0.307
Relative Error of Time (%)	16.08 ± 31.13	21.05 ± 38.90	42.34 ± 49.56	3.985	0.022* ^a^
EMG Burst (mV*ms)	6.396 ± 3.262	5.77 ± 3.657	7.771 ± 4.833	2.310	0.105

Significance indicated by * p < 0.05, ** p < 0.01, and *** p < 0.001.

Abbreviations: EMG = electromyography; ms = milliseconds; N = newtons; PD = Parkinson’s disease; RBD = Rapid Eye Movement Behavior Disorder; SD = standard deviation; mV = millivolts.

Pairwise post-hoc analysis when p ≤ 0.05 (Tukey-corrected):

a = Controls versus PD.

b = Controls versus RBD.

c = RBD versus PD.

**Table 4:** RBD_Mild vs RBD_Moderate Demographics for the Finger and Ankle Studies. Demographics of controls, RBD MDS-UPDRS score under 8 (RBD_Mild), RBD MDS-UPDRS score 9 or higher (RBD_Moderate) and PD. One-way ANOVA and Fisher’s exact test were performed for quantitative and categorical characteristics, respectively, to compare if all groups were similar or different from each other.

Finger Force Study	Controls (n = 27)	RBD_Mild (n = 19)	RBD_Moderate (n = 18)	p-value

Age (years)	62.85 ± 7.735	59.53 ± 10.16	62.61 ± 6.878	0.370
Sex M / F	14 / 13	13 / 6	12 / 6	0.557
Handedness L / R	6 / 21	0 / 19	3 / 15	0.073
Disease Duration (months)	–	17.42 ± 24.17	38.35 ± 50.65	–
MOCA	26.89 ± 2.501	25.68 ± 2.945	25.389 ± 2.725	0.144
MDS-UPDRS-III	3.519 ± 2.327	4.474 ± 2.932	17.778 ± 8.855	< 0.001*** ^a,c^
RBDSQ	1.259 ± 1.318	7.263 ± 2.621	7.667 ± 2.169	< 0.001*** ^a,b^
Ankle Force Study	Controls (n = 26)	RBD_Mild (n = 19)	RBD_Moderate (n = 18)	p-value
Age (years)	63.23 ± 7.628	59.53 ± 10.16	62.61 ± 6.878	0.314
Sex M / F	14 / 12	13 / 6	12 / 6	0.613
Handedness L / R	6 / 20	0 / 19	3 / 15	0.065
Disease Duration (months)	–	17.42 ± 24.17	38.35 ± 50.65	–
MOCA	26.81 ± 2.514	25.68 ± 2.945	25.389 ± 2.725	0.187
MDS-UPDRS-III	3.654 ± 2.262	4.474 ± 2.932	17.778 ± 8.855	<0.001*** ^a,c^
RBDSQ	1.308 ± 1.320	7.263 ± 2.621	7.667 ± 2.169	<0.001*** ^a,b^

Significance indicated by *** p < 0.001.

Abbreviations: F = female; MDS-UPDRS-III = Movement Disorders Society - Unified Parkinson’s Disease Rating Scale - Part 3; L = left; M = male; MOCA = Montreal Cognitive Assessment; PD = Parkinson’s disease; RBD = Rapid Eye Movement Behavior Disorder; RBD_Mild = RBD MDS-UPDRS score < 9; RBD_Moderate = RBD MDS-UPDRS score ≥ 9; RBDSQ = REM Sleep Behavior Disorder Questionnaire; R = right, SD = standard deviation

Pairwise post-hoc analysis when p ≤ 0.05 (Tukey-corrected):

a = Controls versus RBD_Mild.

b = Controls versus RBD_Moderate.

c = RBD_Mild versus RBD_Moderate.

**Table 5:** RBD_Mild vs RBD_Moderate: Maximal Voluntary Contraction, Constant Force, Goal-Directed Tasks for the Finger and Ankle Force Studies. ANCOVAs were run on parametric data for the finger and ankle studies. Age and sex were included as covariates. The Kruskal-Wallis rank sum test was run on non-parametric data (± 1 SD) for the finger and ankle force studies.

Finger Force Study	Controls (n = 27)	RBD_Mild (n = 19)	RBD_Moderate (n = 18)	p-value

*Maximum Voluntary Contraction Task*				
MVC	39.57 ± 21.17	35.97 ± 15.88	35.78 ± 21.44	0.427
*Constant Force Task*				
Mean Force (N)	5.386 ± 2.422	3.702 ± 3.075	4.357 ± 3.860	0.050
CV Force (%)	3.391 ± 1.858	8.682 ± 9.693	11.07 ± 9.907	<0.001*** ^a,b^
RMSE Force (N)	0.352 ± 0.402	1.397 ± 1.989	1.134 ± 1.552	0.060
*Goal-Directed Force Task*				
Peak Force (N)	6.604 ± 3.037	5.599 ± 2.759	6.421 ± 4.098	0.156
Time to Peak of Force (ms)	203.8 ± 34.48	177.2 ± 21.75	207.3 ± 28.57	0.009** ^b,c^
Time to Relaxation of Force (ms)	248.3 ± 48.82	225.3 ± 36.05	257.3 ± 60.05	0.138
Absolute Error of Force (N)	1.531 ± 0.784	1.381 ± 0.759	1.733 ± 1.404	0.340
Absolute Error of Time (ms)	43.39 ± 36.74	34.58 ± 14.25	40.33 ± 15.07	0.590
Relative Error of Force (%)	19.06 ± 17.16	16.90 ± 22.53	20.20 ± 18.64	0.910
Relative Error of Time (%)	9.039 ± 28.98	− 9.556 ± 14.13	8.038 ± 18.59	0.029* ^a^
EMG Burst (mV*ms)	34.69 ± 30.50	31.45 ± 16.39	27.25 ± 24.51	0.600
Ankle Force Study	Controls (n = 26)	RBD_Mild (n = 19)	RBD_Moderate (n = 18)	p-value
*Maximum Voluntary Contraction Task*				
MVC (N)	124.4 ± 46.25	134.0 ± 50.02	136.3 ± 66.35	0.846
*Constant Force Task*				
Mean Force (N)	18.12 ± 6.868	14.80 ± 10.34	17.47 ± 13.79	0.523
CV Force (%)	2.819 ± 1.942	8.427 ± 12.64	11.46 ± 14.60	0.058
RMSE Force (N)	0.979 ± 0.830	5.402 ± 8.390	4.378 ± 6.740	0.004** ^a,b^
*Goal-Directed Force Task*				
Peak Force (N)	24.24 ± 9.248	26.85 ± 12.478	29.11 ± 14.84	0.463
Time to Peak of Force (ms)	204.3 ± 45.73	197.7 ± 41.715	221.3 ± 49.62	0.276
Time to Relaxation of Force (ms)	289.9 ± 44.89	286.1 ± 29.68	290.7 ± 30.80	0.957
Absolute Error of Force (N)	6.681 ± 4.817	8.413 ± 6.365	9.722 ± 7.667	0.327
Absolute Error of Time (ms)	46.98 ± 39.50	43.76 ± 28.02	60.03 ± 69.25	0.553
Relative Error of Force (%)	34.51 ± 34.21	33.60 ± 35.06	42.21 ± 50.45	0.739
Relative Error of Time (%)	16.08 ± 31.13	11.88 ± 27.34	30.73 ± 47.12	0.241
EMG Burst (mV*ms)	6.396 ± 3.262	5.609 ± 2.957	5.941 ± 4.359	0.799

Significance indicated by * p < 0.05, ** p < 0.01 and *** p < 0.001.

Abbreviations: CV = coefficient of variation; EMG = electromyography; MVC = maximum voluntary contraction; PD = Parkinson’s disease; RBD = Rapid Eye Movement Behavior Disorder; RBD_Mild = RBD MDS-UPDRS score < 9; RBD_Moderate = RBD MDS-UPDRS score ≥ 9; RMSE = root mean squared error, SD = standard deviation

Pairwise post-hoc analysis when p ≤ 0.05 (parametric analysis was Tukey-corrected, non-parametric analysis was Bonferroni-corrected):

a = Controls versus RBD_Mild.

b = Controls versus RBD_Moderate.

c = RBD_Mild versus RBD_Moderate.

## Data Availability

The deidentified data and analysis code may be shared upon reasonable request.
